# Supplementary material for: Modeling glioblastoma heterogeneity as a dynamic network of cell states
Source: Mol Syst Biol. 2021 Sep 16;17(9):e10105. doi: 10.15252/msb.202010105 (PMC8444284; doi:10.15252/msb.202010105)
Supplement: Supplementary file 5 — Source Data for Figure 3 [file MSB-17-e10105-s001.zip › Figure3A_sourcedata/GSEA_3065/nosigdb_state6.GseaPreranked.1622718443993/RICHARDS_NOWAKOWSKI_SCIENCE_2017_NIN4_UPREG.html]

Details for gene set RICHARDS\_NOWAKOWSKI\_SCIENCE\_2017\_NIN4\_UPREG[GSEA]

|  || Dataset | state6 |
| Phenotype | NoPhenotypeAvailable |
| Upregulated in class | na\_pos |
| GeneSet | RICHARDS\_NOWAKOWSKI\_SCIENCE\_2017\_NIN4\_UPREG |
| Enrichment Score (ES) | 0.4006962 |
| Normalized Enrichment Score (NES) | 1.7679963 |
| Nominal p-value | 0.0 |
| FDR q-value | 0.006443754 |
| FWER p-Value | 0.119 |
Table: GSEA Results Summary

  

Fig 1: Enrichment plot: RICHARDS\_NOWAKOWSKI\_SCIENCE\_2017\_NIN4\_UPREG      
 Profile of the Running ES Score & Positions of GeneSet Members on the Rank Ordered List

  

| PROBE | GENE SYMBOL | GENE\_TITLE | RANK IN GENE LIST | RANK METRIC SCORE | RUNNING ES | CORE ENRICHMENT || 1 | SOX4 |  |  | 3 | 0.375 | 0.0306 | Yes |
| 2 | RPS27 |  |  | 11 | 0.307 | 0.0551 | Yes |
| 3 | CNP |  |  | 18 | 0.288 | 0.0782 | Yes |
| 4 | RPL10 |  |  | 33 | 0.265 | 0.0984 | Yes |
| 5 | RPS3 |  |  | 59 | 0.238 | 0.1151 | Yes |
| 6 | FTL |  |  | 64 | 0.234 | 0.1340 | Yes |
| 7 | SOX11 |  |  | 70 | 0.230 | 0.1524 | Yes |
| 8 | JUND |  |  | 75 | 0.226 | 0.1706 | Yes |
| 9 | MYL6B |  |  | 83 | 0.223 | 0.1882 | Yes |
| 10 | RPS11 |  |  | 85 | 0.221 | 0.2063 | Yes |
| 11 | PTMA |  |  | 94 | 0.216 | 0.2233 | Yes |
| 12 | RPS23 |  |  | 99 | 0.213 | 0.2404 | Yes |
| 13 | GLCCI1 |  |  | 101 | 0.213 | 0.2578 | Yes |
| 14 | HNRNPA1 |  |  | 108 | 0.209 | 0.2744 | Yes |
| 15 | ASAP1 |  |  | 141 | 0.196 | 0.2868 | Yes |
| 16 | TMSB4X |  |  | 162 | 0.187 | 0.3000 | Yes |
| 17 | OAZ2 |  |  | 201 | 0.176 | 0.3101 | Yes |
| 18 | RPL23A |  |  | 246 | 0.163 | 0.3184 | Yes |
| 19 | CORO1C |  |  | 345 | 0.145 | 0.3190 | Yes |
| 20 | SRP9 |  |  | 376 | 0.139 | 0.3271 | Yes |
| 21 | HNRNPH1 |  |  | 378 | 0.139 | 0.3384 | Yes |
| 22 | HIPK2 |  |  | 445 | 0.128 | 0.3414 | Yes |
| 23 | FBXW7 |  |  | 498 | 0.124 | 0.3456 | Yes |
| 24 | CAMTA1 |  |  | 611 | 0.113 | 0.3420 | Yes |
| 25 | BIRC2 |  |  | 644 | 0.111 | 0.3474 | Yes |
| 26 | SH3BGRL3 |  |  | 700 | 0.106 | 0.3498 | Yes |
| 27 | NFE2L2 |  |  | 748 | 0.102 | 0.3528 | Yes |
| 28 | PCNP |  |  | 763 | 0.101 | 0.3595 | Yes |
| 29 | GFPT1 |  |  | 773 | 0.100 | 0.3667 | Yes |
| 30 | SLC29A4 |  |  | 780 | 0.100 | 0.3743 | Yes |
| 31 | RAB13 |  |  | 858 | 0.094 | 0.3731 | Yes |
| 32 | PFN2 |  |  | 942 | 0.089 | 0.3709 | Yes |
| 33 | COPS8 |  |  | 975 | 0.087 | 0.3743 | Yes |
| 34 | TOX3 |  |  | 999 | 0.086 | 0.3787 | Yes |
| 35 | HMGN1 |  |  | 1060 | 0.082 | 0.3786 | Yes |
| 36 | MARCKS |  |  | 1081 | 0.081 | 0.3830 | Yes |
| 37 | WDR48 |  |  | 1083 | 0.081 | 0.3896 | Yes |
| 38 | NEK1 |  |  | 1085 | 0.081 | 0.3961 | Yes |
| 39 | BBX |  |  | 1139 | 0.078 | 0.3965 | Yes |
| 40 | PBX1 |  |  | 1168 | 0.077 | 0.3996 | Yes |
| 41 | RND3 |  |  | 1213 | 0.075 | 0.4007 | Yes |
| 42 | ANKRD26 |  |  | 1414 | 0.067 | 0.3831 | No |
| 43 | KALRN |  |  | 1543 | 0.062 | 0.3735 | No |
| 44 | RCOR3 |  |  | 1725 | 0.056 | 0.3572 | No |
| 45 | SEPT2 |  |  | 1735 | 0.055 | 0.3607 | No |
| 46 | SLC25A3 |  |  | 1835 | 0.052 | 0.3536 | No |
| 47 | ZBTB44 |  |  | 1941 | 0.049 | 0.3456 | No |
| 48 | CHD7 |  |  | 1953 | 0.049 | 0.3484 | No |
| 49 | FAM76A |  |  | 2078 | 0.046 | 0.3378 | No |
| 50 | CFL1 |  |  | 2433 | 0.035 | 0.2998 | No |
| 51 | DIMT1 |  |  | 2461 | 0.035 | 0.2996 | No |
| 52 | CFDP1 |  |  | 2473 | 0.034 | 0.3011 | No |
| 53 | CWC15 |  |  | 2611 | 0.031 | 0.2878 | No |
| 54 | RAB8A |  |  | 2717 | 0.028 | 0.2781 | No |
| 55 | DCUN1D1 |  |  | 2882 | 0.024 | 0.2612 | No |
| 56 | SF3B2 |  |  | 2889 | 0.024 | 0.2625 | No |
| 57 | MED13 |  |  | 2906 | 0.024 | 0.2626 | No |
| 58 | HSPA4 |  |  | 2968 | 0.023 | 0.2574 | No |
| 59 | DCLK2 |  |  | 3031 | 0.021 | 0.2520 | No |
| 60 | FEZ1 |  |  | 3090 | 0.020 | 0.2470 | No |
| 61 | INTS10 |  |  | 3145 | 0.019 | 0.2423 | No |
| 62 | CPEB4 |  |  | 3220 | 0.017 | 0.2352 | No |
| 63 | GMFB |  |  | 3265 | 0.016 | 0.2314 | No |
| 64 | PGM3 |  |  | 3268 | 0.016 | 0.2325 | No |
| 65 | DENR |  |  | 3311 | 0.015 | 0.2290 | No |
| 66 | YWHAG |  |  | 3404 | 0.013 | 0.2194 | No |
| 67 | EXOSC6 |  |  | 3661 | 0.008 | 0.1905 | No |
| 68 | NCOR1 |  |  | 3732 | 0.006 | 0.1829 | No |
| 69 | FRS2 |  |  | 3764 | 0.005 | 0.1798 | No |
| 70 | PLXNA4 |  |  | 3824 | 0.004 | 0.1733 | No |
| 71 | PCDH17 |  |  | 3912 | 0.002 | 0.1634 | No |
| 72 | UBR1 |  |  | 4106 | -0.001 | 0.1413 | No |
| 73 | FRG1 |  |  | 4308 | -0.005 | 0.1185 | No |
| 74 | TIAM1 |  |  | 4472 | -0.008 | 0.1004 | No |
| 75 | MARK3 |  |  | 4659 | -0.012 | 0.0799 | No |
| 76 | FBXO21 |  |  | 4718 | -0.013 | 0.0743 | No |
| 77 | GAD1 |  |  | 4858 | -0.016 | 0.0595 | No |
| 78 | SLX4IP |  |  | 5174 | -0.022 | 0.0250 | No |
| 79 | SACS |  |  | 5232 | -0.023 | 0.0202 | No |
| 80 | CEP78 |  |  | 5239 | -0.023 | 0.0214 | No |
| 81 | EPHA4 |  |  | 5330 | -0.025 | 0.0131 | No |
| 82 | PLS3 |  |  | 5389 | -0.026 | 0.0086 | No |
| 83 | ACTB |  |  | 5477 | -0.028 | 0.0008 | No |
| 84 | NDUFS1 |  |  | 5530 | -0.029 | -0.0028 | No |
| 85 | DDHD1 |  |  | 5578 | -0.030 | -0.0057 | No |
| 86 | PSMD7 |  |  | 5599 | -0.031 | -0.0055 | No |
| 87 | PRRC2C |  |  | 5705 | -0.033 | -0.0149 | No |
| 88 | SP3 |  |  | 5802 | -0.034 | -0.0232 | No |
| 89 | EPHB2 |  |  | 6035 | -0.039 | -0.0467 | No |
| 90 | ESCO1 |  |  | 6056 | -0.039 | -0.0458 | No |
| 91 | MFN1 |  |  | 6092 | -0.040 | -0.0466 | No |
| 92 | DLD |  |  | 6247 | -0.044 | -0.0607 | No |
| 93 | NUP43 |  |  | 6257 | -0.044 | -0.0582 | No |
| 94 | POMP |  |  | 6316 | -0.045 | -0.0612 | No |
| 95 | VPS35 |  |  | 6333 | -0.045 | -0.0593 | No |
| 96 | LCOR |  |  | 6441 | -0.048 | -0.0676 | No |
| 97 | WAC |  |  | 6463 | -0.049 | -0.0660 | No |
| 98 | CCNH |  |  | 6539 | -0.051 | -0.0705 | No |
| 99 | SLK |  |  | 6589 | -0.053 | -0.0718 | No |
| 100 | MAGI2 |  |  | 6760 | -0.058 | -0.0867 | No |
| 101 | KIFAP3 |  |  | 6776 | -0.058 | -0.0836 | No |
| 102 | PFN1 |  |  | 6817 | -0.059 | -0.0834 | No |
| 103 | CDK5RAP3 |  |  | 6851 | -0.060 | -0.0822 | No |
| 104 | TNRC6A |  |  | 6854 | -0.060 | -0.0775 | No |
| 105 | MRPL39 |  |  | 6858 | -0.061 | -0.0728 | No |
| 106 | EXOC1 |  |  | 7139 | -0.069 | -0.0994 | No |
| 107 | ELOVL5 |  |  | 7151 | -0.069 | -0.0950 | No |
| 108 | HLTF |  |  | 7235 | -0.072 | -0.0986 | No |
| 109 | SEMA6D |  |  | 7344 | -0.076 | -0.1048 | No |
| 110 | MCMBP |  |  | 7507 | -0.082 | -0.1168 | No |
| 111 | WLS |  |  | 7516 | -0.082 | -0.1110 | No |
| 112 | AHCTF1 |  |  | 7610 | -0.087 | -0.1146 | No |
| 113 | SOX6 |  |  | 7616 | -0.087 | -0.1080 | No |
| 114 | TMEM41B |  |  | 7747 | -0.093 | -0.1153 | No |
| 115 | TMEM123 |  |  | 7786 | -0.095 | -0.1119 | No |
| 116 | PPFIA1 |  |  | 7796 | -0.095 | -0.1051 | No |
| 117 | ANKRD11 |  |  | 7843 | -0.098 | -0.1023 | No |
| 118 | SMIM7 |  |  | 7948 | -0.104 | -0.1057 | No |
| 119 | DDX46 |  |  | 7980 | -0.106 | -0.1006 | No |
| 120 | TMTC3 |  |  | 8005 | -0.107 | -0.0945 | No |
| 121 | ZNF148 |  |  | 8078 | -0.112 | -0.0936 | No |
| 122 | TRAM1 |  |  | 8301 | -0.132 | -0.1083 | No |
| 123 | OCIAD1 |  |  | 8423 | -0.149 | -0.1099 | No |
| 124 | TMEM245 |  |  | 8479 | -0.157 | -0.1033 | No |
| 125 | NDFIP1 |  |  | 8490 | -0.159 | -0.0914 | No |
| 126 | PCM1 |  |  | 8496 | -0.160 | -0.0787 | No |
| 127 | AKAP12 |  |  | 8586 | -0.185 | -0.0738 | No |
| 128 | SNTG1 |  |  | 8713 | -0.251 | -0.0677 | No |
| 129 | CANX |  |  | 8735 | -0.270 | -0.0478 | No |
| 130 | APLP2 |  |  | 8755 | -0.292 | -0.0259 | No |
| 131 | MEG3 |  |  | 8786 | -0.370 | 0.0012 | No |
Table: GSEA details [plain text format]

  

Fig 2: RICHARDS\_NOWAKOWSKI\_SCIENCE\_2017\_NIN4\_UPREG: Random ES distribution      
 Gene set null distribution of ES for **RICHARDS\_NOWAKOWSKI\_SCIENCE\_2017\_NIN4\_UPREG**
